# Evaluation of Urban and Rural Family Physician Program in Iran: A Systematic Review

**Published:** 2019-03

**Authors:** Jamaleddin KHEDMATI, Majid DAVARI, Mohsen AARABI, Fatemeh SOLEYMANI, Abbas KEBRIAEEZADEH

**Affiliations:** 1. Department of Pharmacoeconomics and Pharmaceutical Administration, School of Pharmacy, Tehran University of Medical Sciences, Tehran, Iran; 2. Department of Epidemiology, Mazandaran University of Medical Sciences, Mazandaran, Iran

**Keywords:** Family physician program, Family practice, Referral system, Iran

## Abstract

**Background:**

Global experience as well as expert views weight the Family Physician program (FPP) as a primary solution for various problems of healthcare system in Iran. In spite of the valuable information has been collected during conducting FPP, few studies have been done to evaluate the actual performance of this program. This study reviewed the studies related to the evaluation of the FPP systematically.

**Methods:**

The authors systematically searched PubMed, Web of Science, Scopus, Embase, Irandoc and SID for articles published in English and Persian until Nov 2017 without limitation for starting time. Selection stages of the articles were done based on PRISMA flow diagram guidelines.

**Results:**

Of all articles evaluated, 19 were selected. Four articles were removed due to inadequate quality of the study. Only one article evaluates urban and the rest are about rural. Eight articles were categorized as the process evaluations and 12 outcome assessments (one of them was common).

**Conclusion:**

We achieved three main findings. First, the rural FPP has improved access to the healthcare services, but improvement in patient finding and quality of cares remains questionable. Second, there are considerable concerns in the referral system between levels I and II in both urban and rural programs. Third, there was no efficient planning to implement the FP as the gatekeepers of health care system effectively. These issues deprived the efficiency aim of FPP and need serious consideration.

## Introduction

The Family physician (FP) is at the center of all efforts to improve quality, effectiveness, equity, and efficiency in the healthcare systems worldwide (1). A family physician is a community-oriented doctor who is responsible for the care of patients with nonspecific problems. In addition, to have higher efficiency and effectiveness, health systems are organized in a vast majority of countries in a way that enables financially privileged people to have access to specialized services through a referral system (2). Many countries with a national medical system have found that a comprehensive family physician program (FPP) is the most appropriate strategy to achieve effectiveness, efficiency, and equity (3).

FPs as the heart of the family medicine. have a crucial role and act as a communicational bridge between those using health care system and the health care system itself in providing health care services efficiently, and equitably (4, 5).

The establishment of Health Care Networks in the mid-1980s has been one of the remarkable healthcare reforms within the Iranian healthcare system (6). This foundation has improved primary health care (PHC) service and consequently health indicators in Iran significantly (7). The health houses, as the basic building blocks for the Health Care Networks, were settled in rural areas of Iran with less than 5,000 inhabitants. Health centers and health houses provide primary health services to rural residents. First-line health service providers were selected from local residents called Behvarz. A general physician is based in the rural health center; he supervises the center activities and patients visits referred by these community health workers (6).

The existing gap between health care utilization in urban and rural populations was the main reason for implementing another health system reform by the name of family physician program (FPP) and rural health insurance plan for rural inhabitants in Jun 2005 (6, 8). Over a six months period, several family physicians and midwives were employed in health centers to provide services to rural, tribal, and urban areas with a maximum population to 20,000. The main purpose of this effective health sector program was maintaining and promoting the PHC program achievements (6).

Global experiences, as well as expert views, have deemed the FPP as a primary step for managing many challenges of healthcare system in Iran (9). The FPP and rural health insurance plan for rural inhabitants was developed in June 2005, this plan has been considerably successful. Remarkable results of the preliminary phases of the plan caused it to be implemented in 2010 on a pilot basis in 17 cities with a population of under fifty thousand (8). The urban FPP has been implemented in two provinces of Mazandaran and Fars in the cities with a population exceeding twenty thousand since late 2012 (10–12). In the meanwhile, the Ministry of Health and Medical Education (MOHME) had been committed to the full implementation of the nationwide family physician program by the end of the fourth development plan but as a result of budget issues and deficits, this initiation has been postponed (11).

The ‘Family Physician Program’ has launched since 2005 and valuable data are now available about the program outcomes through vital horoscopes. Several studies are conducted in order to evaluate the performance of the program from various perspectives (13–16). The purpose of this study was to review systematically these studies conducted on the evaluation of the FPP.

## Methods

In this systematic review, electronic databases including Embase, PubMed, Scopus, and Web of Science were searched with appropriate keywords. Likewise, Persian equivalents of these keywords were used to conduct search within the Iranian databases of Scientific Information Database (SID), and Irandoc. The search was done in Nov 2017 without imposing any publishing date criterion. We used the following key words in our search strategy: evaluation/ assessment/ impact/ affect, family medicine/ family physician/ family practice/ referral system, and Iran in Title or Abstract.

Studies were selected for inclusion if they met the following criteria:

1. Evaluating family physician program
2. Studies were conducted in Iran
3. Studies were written in English or Persian language On the other hand, the articles which had not even one of the inclusion criterion evaluated pilot plan and evaluated program satisfaction, were excluded.

According to what WHO has mentioned as a philosophy of family physician deployment including quality, effectiveness, equity, and cost reduction, despite importance of stakeholders’ satisfaction, articles that assessed program satisfaction have not been considered.

**Fig. 1:**
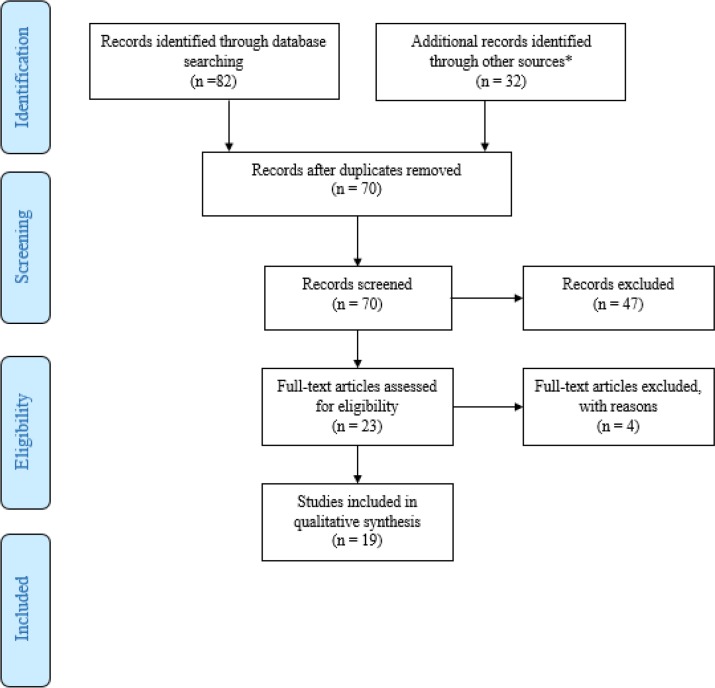
PRISMA Flow diagram

The selection of articles was done by two researchers independently; disagreements were resolved by discussion with the third person. The selection stages were done based on Preferred Reporting Items for Systematic Reviews and Meta-Analyses (PRISMA) guidelines for a systematic review (17) (Fig. 1).

A data extraction form was developed in order to categorize the results and extract them appropriately. The elements of this form were including article specifications (title and authors), study location (rural/urban), assessment method (process/outcome), type of study, time of study, measures and the results. Authors of the articles without enough explanation about measures were contacted by email and/or phone.

## Results

One hundred and fourteen articles were found from databases; 82 from international databases and 32 from Persian websites. After removing the duplications, 70 articles remained for screening.

Next, the articles were evaluated by their titles and abstracts. Twenty-three studies were selected in this step. Four other articles were also removed due to insufficient explanation of measures, plan assessment in pilot cities, and inadequate quality of the study and arguments. The remaining nineteen articles were divided into two groups based on their evaluation method. The summary of the results is presented in Tables 1 and 2. Only one article was evaluated urban FPs and the rest were evaluated rural FPP.

**Table 1:** Data extract of process evaluation studies

***#***	***Authors***	***Type of study and Sampling***	***Location***	***Time of study conduction***	***Measures & Result***
1	Jahromi, V. K. et al.	Cross-sectional study Multistage stratified cluster sampling In Fars and Mazandaran provinces	Urban	Between September 2015 and March 2016	Continuity of Care: Informational, Interpersonal, Longitudinal Computer use 43.3%, No software program Referral letter 88% Feedback 57% 29% of FP know past medical history Visit frequency: FP> health care team
2	Khayyati, F. et al.	Before and after study Randomly selected from health units under family physician program coverage In Sanandaj	Rural	2004 and 2008	Physicians presence: 75 %(2004)-100 %(2008) Midwives presence: 50 %(2004) - 100 %(2008) Referral cases: 2676 cases, 36% follow up rate: 0 %(2004) - 3.17 %(2008) Insurance coverage: 27%(2004) - 97%(2008)
3	Nasrollahpour Shirvani, D. et al.	Cross-sectional study Systematic cluster sampling In northern provinces	Rural	2008	Referral form from health houses: 40.5% // Requests refferal: 46% Defines the level II specialist: 32.9% Relation between specialty and feedback: Yes Rate of return to level I: 24.5% Rate of follow up call by FP and Behvarz: PF 4.8%- Behvarz 14%
4	Nasrollahpour Shirvani, S. D. et al.	Cross-sectional study Randomly selected In northern provinces	Rural	autumn of 2012 and winter of 2013	Referral form from health houses: M: 12%, Gil: 47%, Gol: 46% Requests refferal: M: 28%, Gil: 40%, Gol: 28% Defines the level II specialist: M: 15%, Gil: 19%, Gol: 17% Feedback submission: M: 16%, Gil: 25%, Gol: 10% Rate of return to level I: M: 12%, Gil: 23%, Gol: 13% Recording of refferal results: M:36%, Gil: 79%, Gol: 25% Rate of follow up call by FP and Behvarz: FP: M:1.3%, Gil: 2.4%, Gol: 2.7% Behvarz: M:4.7%, Gil:10%, Gol: 12%
5	Nasrollahpour Shirvani, D. et al.	Cross-sectional study Systematic cluster sampling In IUMS	Rural	2009	Referral form from health houses: 34.7% Requests refferal: 64.9% Defines the level II specialist: 28% Rate of feedback: 30.2%
6	Chaman, R. et al.	Cross-sectional study census sampling method In Shahroud county	Rural	2010	Referral form from health houses: 34.1% Requests refferal by FP: 56.2% Defines the level II specialist: 34% Relation between specialty and feedback: NO Rate of feedback from specialist: about 50% Recording of referral results by FP: 12.8% Rate of follow up call by FP and Behvarz: PF 6.2% – Behvarz 24.1%
7	Ebrahimipour, H. et al.	Cross-sectional study census sampling method In Bardaskan county	Rural	2009–2011	Visit of doctor and midwife: FP: 8.2%–13.7%. MW: 1%–1.6% Referral: In optimal range Use of labs services: over the desired levels Use of radiology services: within the normal range Feedback: low levels of performance as well as patients’ lack of information
8	Dehnavieh, E. et al.	Cross-sectional study	Rural	2014	Referral form from health houses: 26% Requests referral by FP: 56.4% Rate of feedback from specialist: 34%

**Table 2:** Data extract of outcome evaluation studies

***#***	***Authors***	***Location***	***Type of study and Sampling***	***Time of study conduction***	***Measure & Result***
1	Khadivi, R. et al.	Rural	Cross-sectional census method In Isfahan county	2011	Hypertension and DM Case finding: Hypertension: 31.5% DM: 55%
2	Khayyati, F. et al.	Rural	Before and after study Randomly selected from health units under family physician program coverage in Sanandaj	2004 and 2008	Accessibility: FPP had a positive effect on the health services accessibility Case finding: FPP had no effect on case finding
3	Naderimagham, S. et al.	Rural	Time-series analysis Data were gathered from vital horoscopes	1995–2011	Neonatal (NMR), infant (IMR), and under-5-year (U5MR) mortality rates in rural areas: NMR and IMR decreased statistically significant. U5MR decreased statistically non-significant
4	Raeissi, P. et al.	Rural	Quasi-experimental before-after study	2001–2004 and 2005–2007	Rate of stillbirth: Decreased, non-significant Percentage of births weighing less than 2500 grams: increased, non-significant Neonatal mortality rate: Decreased, non-significant Under 5-mortality rate: Decreased, non-significant Maternal mortality rate: Decreased, non-significant Under 5-mortality rate due to diarrhea: Decreased, non-significant Under 5-mortality rate due to respiratory infection: Decreased, significant Infant mortality rate: Decreased, significant
5	Jabbari-birami, H. et al.	Rural	Cross-sectionalcensus and randomized cluster samplingIn East Azerbaijan	2000 – 2006	Birth rate: steady state Family health services coverage: 39.7% to 66.2% Using Contraceptive injection: 1% increased Using condom: 10% increased Using OCP: 4% decreased Using IUD: 6% decreased Periodical checkup: 92.6% to 98.3% Pap Smear test: 14.1% to 76.4%
6	Alipour, A. et al	Rural	Cross-sectional Census method In Sari county and Soorak city	2003–2007	1. Family planning Condom: 5.71% increased significant Injection: 1.19% increased non-significant vasectomy: 0.03% increased non-significant Tubectomy: 3.95% Decreased significant Traditional: 2.56% Decreased significant IUD: 0.29% Decreased non-significant Norplant: 0.02% Decreased non-significant OCP: 1.35% Decreased non-significant
7	Kazemian, M. Kavian-Telour, F	Rural	Descriptive - Analytic study Registered data from in 17 rural health centers in the Gorgan province	2011–2012	Access to Health Care: Increase Comprehensive Health Care: Physician share is much more than other services (nurses, lab, pharmacy)
8	Kazemian, M. et al.	Rural	Descriptive - Analytic Registered data from in 17 rural health centers in the Gorgan province	2011–2012	10% increasing in monthly comprehensive care per capita decreases the unnecessary specialist visit: Physician: 6.6% Nurse: 0.2% pharmacy:1.2% lab: 1.7%
9	Khadivi, R. et al.	Rural	Descriptive - Analytic census method In Isfahan	2004–2011	Prescription per capita: 0.145 to 0.64 Average number of prescription items: 4.27 to 4.11
10	Fallah, S Rostamzadeh, S	Rural	Retrospective cohort study Systematic randomized sampling In health centers of Kordkoy		Retinopathy, Nephropathy and Stoke: No significant difference Diabetic foot ulcer: More in control group Heart attack: More in case group
11	Golalizadeh, E. Mousazadeh, M.	Rural	Cross-sectionalcensus method	2004–2009	The average of number of visits: Physician: 0.3 to 1.3 Midwife: 0.06 to 0.4 Lab: 0.2 to 0.58 Pharmacy: 0.03 to 0.08 (non-significant)
12	Rashidian, A. et al	Rural	Interrupted time series monthly hospitalization data from MSIO (Medical Services Insurance Organization) records	2003–2007 (immediate effect) 2012 (long term effect)	Annual hospitalization rates: 2003–2007: 44.3 to 65.6 2012: significant decline

### Process Evaluation Studies

Eight articles were addressed process evaluation in their analysis (Table 1). Jahromi evaluated continuity of care (COC) service in urban family physician in three areas; informational (providing accurate patients information by health care system), interpersonal (patient and health care provider relationship), and longitudinal (providing health care continuously). The following results had been obtained: Computer was used without software (43.3%) program or with poor capability (31.9%). About 88% of referred patients to specialist had referral letters and 57% of patients got feedback from specialists. In addition, 29% of FPs had past medical history of patients. Most patients declared they had good relations with their doctors. In general, the results of interpersonal continuity measures of Mazandaran were better than Fars. The frequency of FPs visits was higher than other health care team members, in both provinces. Consequently, there are some problems with three levels of COC in urban FPP (16).

In the study conducted in four health centers and two health houses in Sanandaj, the presence of physicians and midwives were 75% and 50% in 2004 respectively and 100% for both in 2008. In the winter of 2008, 12.3% of the visited patients were hospitalized and 36% of the referred patients were taken feedback and 2.17% of the patients were followed up by the physicians. While in 2004, none of these parameters even existed. There is no significant difference in case finding and referrals from health houses to health centers among these two years, but insurance coverage rose from 28.3% to 97.5% (18).

The referral system was studied in the rural FPP in the Northern provinces of Iran in 2008 and found out that first 40.5% of the patients referred to level II by family physicians had the referral form from the health houses. Second, 46% of these patients were referred based on the family physicians’ judgment and 54% were referred due to the patients’ request. Third, family physicians were engaged in choosing the level II specialist doctors only in 32.9% of the cases; in the rest of the cases, the patients were selected the specialists based on their own decisions. Fourth, of all the patients referred to level II, only 17.6% submitted feedback to the respective health houses. Fifth, family physicians made follow up calls in only 4.8% of the cases and their assistants (Behvarz) made merely 14% follow up calls. Many of the referral system rules failed to meet their primary expectations (15).

Many factors were evaluated including the referral of the patients to family physicians from health house, the need to be referred to level II health care services, the rate of specialist selection by family physicians, the rate of feedback submission, and the rate of referring back to FP. The summary of their results suggested that considerable number of rules and regulations of FP program were ignored or done poorly (19). This statement is also supported by another (20). A cross-sectional study was conducted to evaluate referral system in rural FPP. The results showed 26% referred from health houses by referral form, 56.4% referred to specialists by FPs. Feedback rate by specialists was 34% due to patients’ lack of knowledge. Quality of referral system was not satisfying (13).

The referral system was studied in all the health centers in Shahrood using the questionnaire which validated by Shirvani. 34.1% of the cases referred to family physicians from health houses and the rest had done it on their own. Family physicians decided referral necessity in 56.2% of all cases and chose specialists in 34% of all cases. No significant relation was observed between the feedback level and the specialty of the doctors. About half of level II doctors provided no feedback to family physicians. Records of received healthcare services were registered in patients’ files in 12.8% of patients returning to family physicians. Follow up during the referral period was 6.2% by physicians and 24.1% by Behvarz (21). The performance of FP teams was studied in 7 health centers in Bardaskan between 2009 and 2012. Based on the program guidelines, the numbers of referrals were in an acceptable range while specialists feedback were weak due to their low levels of performance as well as patients’ lack of information. The numbers of laboratory tests were over the desired levels and radiology services requests were within the normal range (22).

### Outcome Evaluation Studies

Hereinafter, outcome evaluation articles are reviewed and explained. The summary of these 12 articles is showed in Table 2. Khadivi et al. assessed the case finding of FPs in Isfahan. The result showed a significant increase in the case finding of hypertensive and diabetic patients, based on the national guidelines, after the start of the rural FPP (23).

A time series analysis was conducted about child mortality including neonatal mortality rate (NMR), infant mortality rate (IMR), and under-five mortality rate (U5MR) in Vital Horoscope between the years 1995 – 2011. Child mortality went down during that period and the FPP had a significant effect on the NMR and IMR parameters but the decrease in U5MR was not related to this program statistically (24).

The effect of the FPP on mother and child health parameters was studied in the years 2001–2005, and 2005–2007 on mothers and children under the coverage of health houses and health centers of the Mashad University of Medical Sciences. Although no significant difference can be identified in the parameters before and after the plan, the trend shows the positive effects of the program (25).

Birth rate and family health services coverage were studied in three cities, Osko, Khosroshahr, and Gogan, of Eastern Azerbaijan province. The results of the study are as follows: There is no considerable change in the birth rates. At the same time, there were four physicians and four midwives before the plan and were doubled after the program started, and the use of condoms and hormone injection have increased by 10% and 1% respectively; while, contraceptive pills, and IUD have decreased, the former by 4% and the latter 6%. The average age of the family size, as well as women’s ages in the years after the start of the program, is lower than before the program which is statistically significant. Unplanned pregnancies in both periods were only one case which is not considerable. Pap smear and Periodical checkups of women under coverage of centers rose significantly from 4.1% to 76.4% and 92.6% to 98.3% respectively. Before the FPP, 92.3% of women and after the initiation of the program, 94.7% of them were under complete coverage of family healthcare services all year around. Generally, findings indicate relative changes in the number of services with not much difference in the quality (14).

The effect of FPP on birth control methods was studied in 27 rural healthcare centers in Sari County and healthcare center of Soorak city using census methods based on Vital Horoscope. Time trend of family control program methods usage was evaluated and the following results were provided. The application of condom, hormone injection, vasectomy and total modern contraceptives increased, whereas using the methods of tubectomy, IUD, OCP, Norplant and traditional method were decreasing. These changes just for condom, tubectomy and traditional methods were statistically significant. Family physician program was relatively successful in family planning (26).

Kazemian et al. used the collective registered data of rural and urban areas with under 20,000 inhabitants of Jalin and Sarkhankalateh in Gorgan in 2012 and 2013. After the assessment of access to outpatient treatment services with the approach of comprehensive care, the access has been increased; also, the share and influence of physician in comprehensive care in the program is still much higher than that of nurses, pharmacies, and labs (27).

Other study in the same areas in 2012 and 13 over a 24 months’ period, the decrease was determined in unnecessary costs of specialist doctor services with the comprehensive care approach of visiting family physicians. A 10% increase in visiting family physicians results in 6.6% cost reduction of unnecessary referrals to specialists and a 10% increase in referring to nurses, pharmacies, and labs results in 0.2%, 1.2%, and 1.7% cost reduction respectively caused by unauthorized referral to specialists (28).

Khadivi et al. studied the consumption of medicine based on the prescription of physicians of the family physicians program and medicinal centers of rural healthcare centers of Isfahan and found out the following results: Annual prescription per capita in 2005 and 2012 was 0.145 and 0.64 respectively. The average number of items per prescription was 4.27 in 2005 and 4.11 in 2012. They concluded that seemingly, in line with increasing number of annual prescriptions per capita, access to physicians services have increased. In addition, with fewer items per prescription, prescribing could be more rational (29). Fallah et al. conducted a retrospective cohort study in the rural health centers of Kordkoy County on the diabetic type II patients with at least 3 years history of diagnosis who exhibited no initial complications upon diagnosis and found out the following: There is no significant difference in retinopathy, nephropathy, and stroke among the control and case groups. Diabetic foot ulcer was higher in the control group and heart attack was higher in the case group. The FPP of Kordkoy Country has not been successful in prevention, reduction, or delaying of the short and long-term complications of diabetes type II (30).

Golalizadeh and Moosazadeh studied on effect of the FPP on the number of visits to all the centers of the healthcare network under the coverage of Mazandaran University of Medical Sciences. They used the census data between the years 2005 and 2010 and found out the following: Average of number of visits to physicians, midwives, medical labs, and pharmacies were 0.3, 0.06, 0.2, and 0.03 before the FPP and 1.3, 0.4, 0.58, and 0.08 respectively after the start of the program. Number of visits to physicians, midwives, medical labs, and pharmacies have increased after the start of the plan, but this increase has not been significant in the case of pharmacy visit (31).

The hospitalization rate of patient covered by MSIO (Medical Services Insurance Organization) evaluated in Lorestan Province in time series. Results showed the annual hospitalization rate increased from 44.3 to 65.6 per 1,000 inhabitants from 2003 to 2007. In the case group, they found that after 40 months of starting the intervention, a new interruption was occurred, which is declining significantly in hospitalization and returning to the trend of pre-plan. These findings suggest, “The increase in the hospitalization rate that observed in the rural population reflects a preexisting unmet need for hospitalizations” (9).

## Discussion

The aim of this study was to review the studies on evaluation of the FPP systematically. Though more than 5 years have been passed since the launch of the urban FPP, only one article has evaluated this program, determined the existence of some shortcomings in the field of information and interpersonal continuity of care (16). However, 18 studies were evaluated rural FPP.

Khadivi et al. have found that the FPP was successful in finding diabetic and hypertensive patients whereas it was ineffective in case finding (18, 23).

Of the five studies conducted using the same methodology on the referral system, two have been done in different times in the Northern cities, one in an area under the coverage of the Iran Medical Science University, one in Shahrood and one in Jiroft; all have admitted the serious issues with the referral system. One of the functions of a family physician is to act as the gatekeeper for access to specialized care, which has obviously not practiced properly due to the level of referrals done by the insistence of patients (32). In addition, as determined in all selected studies, there is very little feedback from higher levels of care to family the physicians, which in turn affects patients’ follow up and consequently leaves family physicians unaware of the result of their referrals. In cases where patients require continuous disease management, quality of care will decrease due to family physicians’ lack of necessary information (13, 15, 19–21).

In accordance with above studies, Ebrahimipour et al. have stated that feedback function was performed poorly in FPP (22). This finding was confirmed later by Dehnavieh et al. study (13).

Two other studies have identified an improving trend in pediatrics and children’s health and confirmed the positive effects of the program (24, 25). Nonetheless, one of these studies stated that the improvement trend was not significant (25). In studies of the FPP’s influence on birth control, relative success was observed. Of course, women’s gynecology visits was increased which was a positive point of the program (14).

Two studies with concentration on comprehensive care in Gorgan County were shown that the FPP had increased the accessibility of outpatient healthcare services; with increasing the number of annual visit to physicians, nurses, medical labs, and pharmacies. They illustrated that the cost of unnecessary visits to specialists were decreased and then the program was highly effective and cost-saving among the services (27, 28).

Khadivi et al. have studied the difference in annual prescription per capita before and after the FPP and have found it to be significant. They showed that prescription pattern has improved through a decrease in the number of items per prescription (29, 31). However, since the number of items per prescription is not enough to evaluate the prescription pattern, it is necessary to undertake further studies to show the effect of FPP on prescription pattern.

A study in Kordkoy County in Iran is showed that, family physicians had not been able to prevent, decrease, or delay the onset of diabetes type II complications (30). But, more studies in different parts of the country are required to evaluate and confirm the conclusion of this study.

Studies that focused on the effect of the FPP on the visits of doctors and hospitalization are confirmed that the FPP has increased accessibility of patients to healthcare services (9, 31). Though the researchers have deemed these increases as the result of unmet needs, but these claims need to be further explored.

## Limitation

This review was done by searching only electronic databases and therefore we may miss the Persian papers which were not indexed in the electronic databases. Nevertheless, because most of the academic journals are publishing electronically, we possibly did not miss very much.

## Conclusion

Access to health care services has been improved with the implementation of FPP; but the referral and communication system between primary and secondary levels of health care services, in both rural and urban area, were not working effectively. Likewise, the main role of FPs as gatekeepers for health care system was not completely implemented. These issues deprived the efficiency aim of FPP and therefore need serious consideration. However, none of our selected articles had been evaluated the efficiency of the FPP in Iran directly.

## Ethical considerations

Ethical issues (Including plagiarism, informed consent, misconduct, data fabrication and/or falsification, double publication and/or submission, redundancy, etc.) have been completely observed by the authors.
